# The Potential Role of Awe for Depression: Reassembling the Puzzle

**DOI:** 10.3389/fpsyg.2021.617715

**Published:** 2021-04-26

**Authors:** Alice Chirico, Andrea Gaggioli

**Affiliations:** ^1^Catholic University of the Sacred Heart of Milan, Milan, Italy; ^2^Applied Technology for Neuro-Psychology Lab, Istituto Auxologico Italiano, Milan, Italy

**Keywords:** awe, mental health, depression, Matryoshka model, complex emotions

## Abstract

Recently, interest in the unique pathways linking discrete positive emotions to specific health outcomes has gained increasing attention, but the role of awe is yet to be elucidated. Awe is a complex and transformative emotion that can restructure individuals' mental frames so deeply that it could be considered a therapeutic asset for major mental health major issues, including depression. Despite sparse evidence showing a potential connection between depression and awe, this link has not been combined into a proposal resulting in specific intervention guidelines. The aim of this perspective was three-fold: (i) to provide a new unifying model of awe's functioning—the Matryoshka model; (ii) to show systematic and explicit connections between this emotion and depression; and (iii) to suggest specific guidelines of intervention utilizing the potential therapeutic role of awe for mental health, specifically for depression. This theoretical endeavor in its entirety has been framed within the health domain.

## Introduction

Recently, within the framework of positive psychology (Ryff and Singer, 2000; Seligman and Csikszentmihalyi, 2000), interest in the unique pathways linking discrete positive emotions to wellbeing promotion has emerged increasingly (e.g., Barrett-Cheetham et al., 2016). Although awe—a complex emotion characterized by an appraisal of vastness and a need for accommodation—has attracted the interest of many researchers in the last 20 years (Sundararajan, 2002; Keltner and Haidt, 2003; Shiota et al., 2007; Chirico et al., 2016; Nelson-Coffey et al., 2019; Chirico, 2020), this phenomenon is still “*in need of research attention in the realm of well-being*” (Barrett-Cheetham et al., 2016, p. 603). This is especially true in the mental health domain.

Pathways between several negative emotions and poor health have been widely investigated (Coifman et al., 2016; Kunzmann et al., 2019), yet the role of specific discrete positive emotions in fostering mental health has only recently become an object of interest (Tugade et al., 2004; Cohen and Pressman, 2006; Anderson et al., 2018; Chirico et al., 2021), especially within the positive psychology framework (Shiota et al., 2017). However, the contribution of discrete complex emotions, such as awe, is practically unexplored. Awe could be considered as an exemplar of this category due to its multi-componential nature stemming from the interaction of simpler emotional aspects, both positive and negative (Grossmann and Ellsworth, 2017). Just its unusual nature would deserve special attention when mental health is involved.

Here, we focused on a specific mental issue whose potential connection to awe has progressively emerged but is still only implicitly discussed and whose relevance has increased and will likely increase in the near future (Frankham et al., 2020; Gunnell et al., 2020): Major Depression Disorder (MDD). We aimed to outline the potential therapeutic role of awe for depression by considering the potential connections between these two phenomena across multiple levels—namely, the psychological, hormonal, neurophysiological, and existential levels. To this end, we provided a preliminary unifying proposal on awe's functioning based on empirical studies addressing the neuro-psycho-physiologic, metabolic, psychological, and existential dimensions of awe. We then drew connections between awe and depression, relying on the same levels. Finally, we combined this evidence into a proposal resulting in specific intervention guidelines for solutions exploiting the therapeutic potential of awe for the depression domain, where scientifically valid accessible and feasible solutions are always needed (e.g., Biddle and Asare, 2011; Bourne et al., 2018).

## A Unifying Proposal on Awe's Functioning: the “Matryoshka” Model

The role of several functionally distinct positive emotions for mental health has been increasingly investigated in the last 20 years (e.g., Tugade et al., 2004; Ong et al., 2018; Chirico et al., 2021), including the depression domain (e.g., Gruber et al., 2009). Awe has acted as a special case. After the seminal theoretical work of Keltner and Haidt (2003), which reported the dimension of *vastness* and *need for accommodation* as the core components of this emotion, several empirical efforts have been devoted to unveiling the potential of awe for human flourishing [for a review, see Chirico (2020)], but the mental health domain has remained nearly unexplored. At the same time, the empirical effort around awe has not been accompanied by an up-to-date unifying proposal on its functioning that is also able to elucidate its unique pathway to health outcomes. Here, using empirical data on awe collected so far, we provided an up-to-date unifying proposal on its fuctioning in the short, medium, and long term in order to elucidate the link between this emotion and Major Depression Disorder as one of the most severe mental issues nowadays.

**Figure 1:**
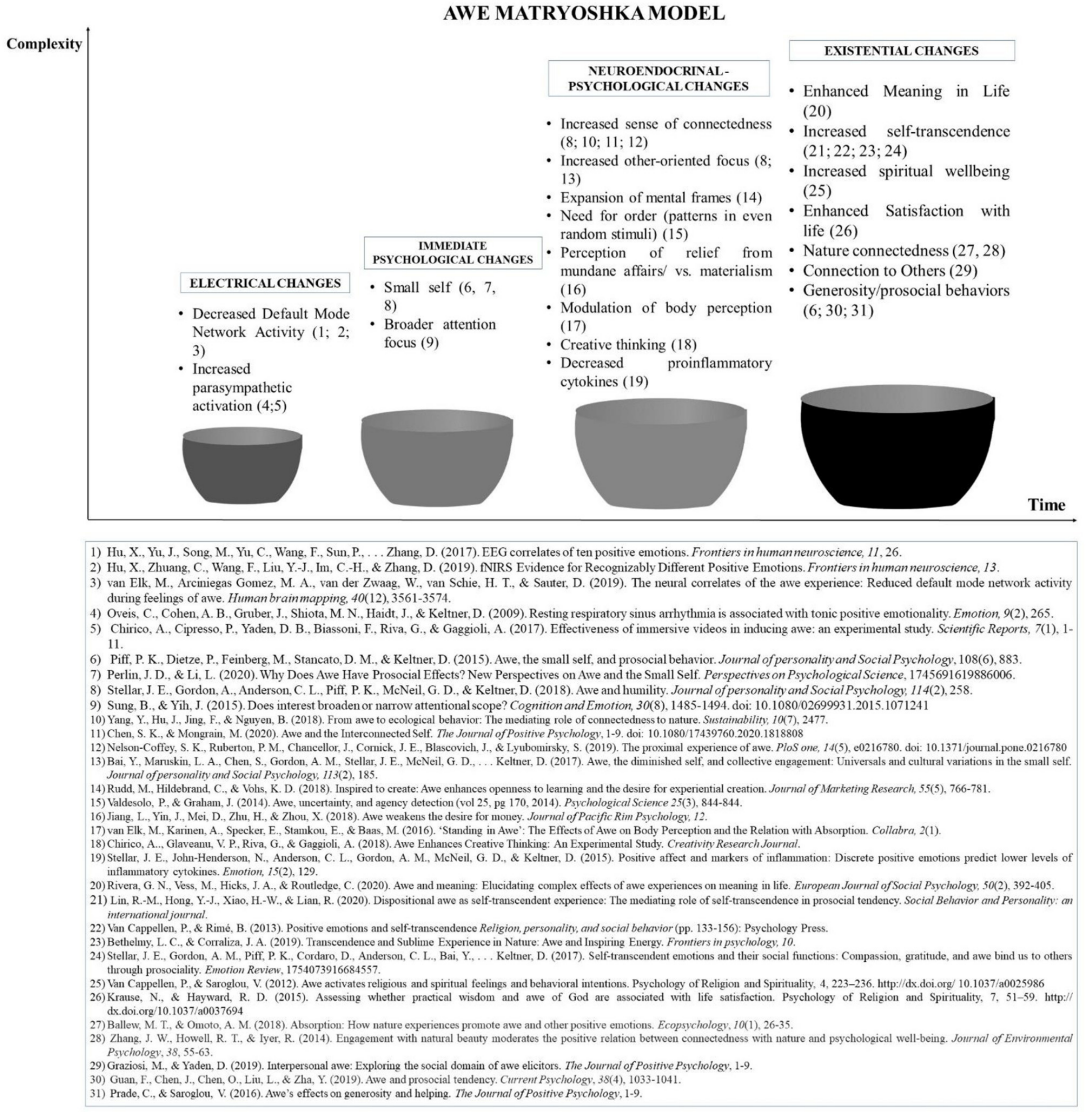
The Matryoshka model.

The potential of this emotion has been deemed to be so vast that it has been considered in relation to a wider process of *transformation* (Pearsall, 2007; Chirico et al., 2018a; Chirico, 2020), or a sudden change after which the person is no longer him-/herself (Skalski, 2009; Paul, 2014; Gaggioli, 2016; Riva et al., 2016); it requires a catalyst, which might be awe (Schneider, 2009; Valdesolo and Graham, 2014; Gaggioli, 2016). This emotion emerged as having a differential impact on individuals as featured by two dimensions: *complexity* of awe-related changes and *time* (i.e., duration or frequency of occurrence). Specifically, empirical studies on this emotion evidenced that awe can affect people by unfolding from a physiological or neurophysiological level, through a psychological one, to an existential one (Schneider, 2009, 2017; Stellar et al., 2015, 2018; Gordon et al., 2016; Hoeldtke, 2016; Bai et al., 2017; Hu et al., 2017). Awe can then act as both a contingent moderately intense phenomenon (Silvia et al., 2015) and a really intense punctual emotion (Chirico et al., 2018a,b) or as a frequent emotional state occurring several times (Shiota et al., 2006; Bonner, 2015; Zhao et al., 2019; Chirico et al., 2021). *The more awe involves increasingly sophisticated changes, the more it evolves toward more complex forms*. To unify all these dimensions of awe, we proposed a synthetic model organizing all of these changes, which we called the “Matryoshka” model, where the most basic levels of awe unfold toward a more sophisticated experience over time (Figure 1).

Awe's *electrical* changes (i.e., DMN activity, parasympathetic, sympathetic activity) would act as mediators between a single awe exposure and its *psychological* (i.e., small self, connectedness, expansion of mental frames, seeking for order, relief from contingencies, meaning in life, broader attention), perceptual (i.e., body perception), and behavioral consequences (i.e., generosity behaviors, helping behaviors, ecological purchase, creative ideas), all tapping into superordinate *existential* processes related to well-being and general health (e.g., satisfaction with life, spiritual well-being, nature connectedness, connection with Others, self-transcendence). Repeated exposure to awe entails the reduction of proinflammatory cytokines—*the neuroendocrinal level*—and this aspect encompasses the whole process of awe, but it becomes evident later.

To show the potential therapeutic role of awe for depression, we showed potential *connections* between this emotion and Major Depression Disorder (MDD), relying on the four previously presented levels of awe's functioning: (i) the electrical level; (ii) the immediate basic psychological level, (iii) the more sophisticated psychological level, which also includes hormonal chemical changes; and (iv) the existential level. We then further clustered the four levels according to their *time occurrence*. First, the neurological and physiological changes associated with awe were reported in the very short term. We then deepened the medium-term changes involving a basic and more sophisticated psychological level as well as hormonal effects. Finally, we introduced and discussed the most complex changes expected in the long run at the existential level.

## Awe and Depression: A *Fil Rouge*?

Depression is a major health problem, with a prevalence of between 8 and 12% worldwide, and it is considered as the second biggest disease burden (Ferrari et al., 2013). Its incidence is assumed to increase in the coming months given the past and actual conditions due to the Covid-19 pandemic (Gunnell et al., 2020), and this needs to be managed in advance (Gunnell et al., 2020).

According to the DSM-5, to be diagnosed as MDD, a person must be experiencing five or more core symptoms (e.g., mood decay most of the day; diminished pleasure; significant weight loss; loss of energy; concentration difficulties; suicidal ideation or thinking of death frequently; feelings of worthlessness; reduced physical movement and slowing down) over a 2-week period, and at least one of the symptoms should be either depressed mood or the loss of interest or pleasure.

The link between awe and depression was elaborated from some core symptoms of MDD. We considered that not all individuals fully meet MDD criteria, thus falling under the threshold of MDD, but they still present difficulties associated with this disease, such as a persistent and relevant decay of moods or a sense of hopelessness (Uher et al., 2014), which, according to our hypothesis, could be ameliorated by awe anyway [see Anderson et al. (2018), Leavell et al. (2019)]. The rationale of this work concerns the potential therapeutic role of awe in contrasting both the symptoms of MDD and some neurophysiological and psychological mechanisms characterizing this pathology.

### An Electrical Connection: The Short-Term Level

#### Neurological Changes

A few studies have begun to shed light on the neurophysiological correlates of awe. Hu et al. (2017) investigated the EEG correlates of ten positive emotions (including awe), which were clustered in three superordinate factors. Awe clustered along with pride, inspiration, hope, and gratitude and was classified as belonging to an “encouragement” factor. Gamma and alpha activity suggested including awe in this cluster as it was positively related to both the central alpha and the beta band and showed negative correlations with the gamma band. The authors explained these findings in terms of an association between these emotions and enhanced cognitive processing. Actually, awe is known to come along with a need to restructure mental schemas—a “*need for accommodation*,” in the words of Keltner and Haidt (2003)—also associated with a deep uncertainty (Valdesolo and Graham, 2014). Other authors have furthered this research by analyzing the activity of a specific brain network, such as the Default Mode Network (DFM) (van Elk et al., 2019), whose increased activity is usually related to increased self-processing and mind-wandering (Qin and Northoff, 2011; Whitfield-Gabrieli and Ford, 2012) and is attenuated during goal-oriented activities (Bressler and Menon, 2010; Menon, 2011). DMN activity decreased during awe-inspiring videos, especially when participants were required to get absorbed passively (van Elk et al., 2019).

The decreased activity of the DMN is a key neural counterpart of MDD, as shown in several works (e.g., Whitfield-Gabrieli and Ford, 2012). Abnormal activity of DFM is associated with more persistent MDD (Li et al., 2013) while increased DFM connectivity (Greicius et al., 2007) is linked to a higher familiar risk for depression (Posner et al., 2016) and generally increases in depressive individuals (Zeng et al., 2012), especially in the anterior portions (Coutinho et al., 2016). Indeed, functional MRI studies have often showed hyperactivity in the amygdala and the ventral components of the anterior cingulate cortex in MDD, which would also play a key role in treatment response (Arnone, 2019).

Moreover, recently, awe has also been proposed as a potential psychological mechanism mediating the effect of psychedelic-occasioned mystical experiences—especially due to psilocybin—on depression (Hendricks, 2018). This link with the psychedelics domain is not new, as Keltner and Haidt described awe as a “psychotic break or a psychedelic experience” (p. 298) when discussing awe in religion. Specifically, at the neural level, psychedelics generate an augmented global functional connectivity and a reduced activity of the DMN, much like the experimentally induced awe. In other terms, psychedelics originate an experience of ego dissolution having a clear overlap with the “small self” experience induced by awe (Hendricks, 2018). Awe may act as a vicarious mechanism modulating the activity of DMN, akin to psychedelics.

#### Psychophysiological Changes

Regarding the peripheral physiological system, preliminary evidence has shown that a single experience of awe was associated with both the withdrawal of a sympathetic system (Oveis et al., 2009) and a parasympathetic activation (Chirico et al., 2017b), as well as with goose bumps (Quesnel and Riecke, 2018). In other words, awe's physiological response resembled a sort of freezing, which resulted in being consistent with evolutionary explanations of this emotion (Keltner and Haidt, 2003; Shiota et al., 2017; Chirico and Yaden, 2018) and its behavioral consequences concerning the perception of the “small self” (e.g., Piff et al., 2015; Stellar et al., 2018; Perlin and Li, 2020).

The ability of awe to modulate cardiac activity can be a key asset for consideration in relation to depression. Indeed, MDD is associated with reduced activity of the vagus nerve (Chang et al., 2012), which affects the overall self-regulation of the organism (Laborde et al., 2018).

### A Psychological and Chemical Connection: The Medium-Term Levels

#### Psychological Changes

At the immediate basic psychological level, awe generates a deep sense of self-diminishment, different from annihilation, where the self is set apart and the attentional focus is oriented “outside” (Sung and Yih, 2015) and above (Yaden et al., 2017). In addition, grief associated with a significant loss by art and nature can be softened if a person experiences awe (Koh et al., 2019). Conversely, patients with MDD reported an increased focus on the self and a decreased one on others, which dons the guise of an uninterrupted negative self-referential thinking associated with dysfunctional regulatory strategies, such as rumination and hopelessness (Nejad et al., 2013).

In this regard, preliminary evidence concerning the potential role of awe in reducing ruminative self-referential tendencies and sense of hopelessness was provided by Tarani (2017), who exposed healthy participants to awe-inspiring vs. amusement-inducing 4-min-long videos. They found that this emotion could decrease two key MDD symptoms: brooding ruminative tendencies (i.e., constant negative self-reflection associated with self-blame) and sense of hopelessness (i.e., expectancy of negative outcomes and helplessness).

Moreover, experimentally induced awe can orient people to interpreting even random events as the result of intentional and purpose-driven agents (Valdesolo and Graham, 2014). For instance, awe prompts us to deal with uncertainty and overcome it by also finding a completely novel explanation (i.e., accommodation). Moreover, awe enables us to broaden our attention focus (Sung and Yih, 2015), thereby facilitating the creation of unprecedent connections among ideas (Chirico et al., 2018b). Awe can even shape people's tendency to support either a scientific or a supernatural explanation of events, depending on their existing levels of theism (i.e., to what extent they believe in God). This cognitive pattern is opposite to the one showed by patients with MDD, who tend to perceive a lack of meaning, order, and purpose in the world (i.e., sense of hopelessness; Abramson et al., 1989). It should be noted that awe and hope fell within the same cluster in Hu et al. (2017).

#### Neuroendocrinal Changes

At the hormonal level, psychoneuroimmunology has drawn upon a different perspective on emotions, which are now considered more than ephemeral phenomena and closer to drivers of our well-being and health. Preliminary evidence suggests that awe is associated with a reduction of proinflammatory cytokines, specifically levels of interleukin-6 (IL-6) (Stellar et al., 2015). From this perspective, living in awe on a daily basis would—alone—be able to shape individuals' physical health at the endocrine level.

On the other hand, peripheral inflammatory states have been found to be associated with central nervous system changes in depression (Peruga et al., 2011; Haji et al., 2012; Lee and Giuliani, 2019). Specifically, an acute increase in pro-inflammatory cytokines produced a sickness syndrome with symptoms overlapping with depression (Capuron et al., 2002). If inflammation contributes to depression (Raison and Miller, 2011), then, interventions targeted at reducing inflammation may act as a preventative measure toward this mental disorder.

### An Existential Connection: The Long-Term Level

At the existential level, both empirical and theoretical evidence supports the self-transcendent nature of this emotion (Van Cappellen et al., 2013; Yaden et al., 2016, 2017, 2019; Li et al., 2019; Kitson et al., 2020). As also reported by the recent work of Chen and Mongrain (2020), awe is a “self-expansive emotion” making us transcend our self. Awe expands our sense of connection with the world (Yang et al., 2018) and other human beings, (Quesnel and Riecke, 2018) thereby making us overcome the sense of loneliness and meaninglessness at the core of MDD (Fried et al., 2015).

Preliminary evidence has already shown a link between self-transcendence and depression (Haugan and Innstrand, 2012). Awe, alternatively conceived as an emotional component of the transcendent experience of the sublime (Bethelmy and Corraliza, 2019; Clewis et al., 2021), as a disposition able to foster self-transcendence meaning in life (i.e., spiritual self-transcendence (Lin et al., 2020), or as a mediator between nature and reduced rumination (Lopes et al., 2020), can always elevate us beyond the limit of our mundane affairs toward something bigger than our self and our concerns (Saroglou et al., 2008; Van Cappellen et al., 2013; Krause and Hayward, 2015). This also provides relief from the constant decay of mood characterizing MDD, and it emerged especially in the field of study related to awe-inspiring natural scenarios (e.g., Anderson et al., 2018).

Finally, the ability of awe to deconstruct existing expectations of the world and others—the “transformative potential” of awe (Chirico et al., 2016)—can act as a counterpart to the major issue of cognitive fixedness in MDD (Kube et al., 2017), as also supported within a recent framework on depression provided by the free-energy theory (Fabry, 2019). Therefore, we speculate that awe's transformative potential may be key in unlocking fixed-expectation processes typical of MDD at different ages (Benzi et al., 2018, 2020, 2021).

### Awe and Depression: Reassembling the Puzzle

**Figure 2:**
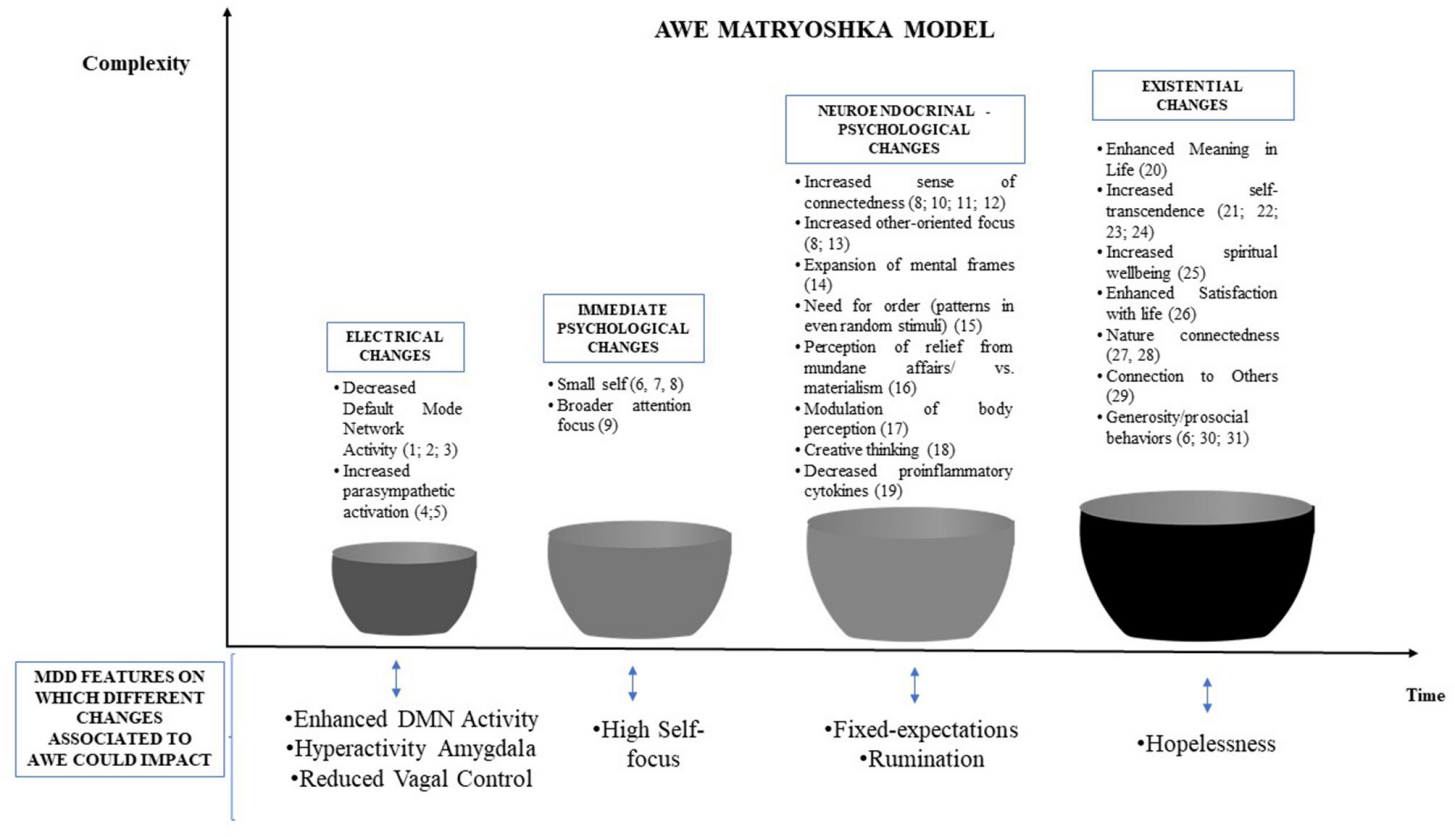
Synthesis of the potential connections between awe and depression.

All the components of awe outlined in Figure 1 can act as counterparts to specific dimensions of depression (see Figure 2 for a graphical synthesis). First, awe can decrease the activity of DMN, which is hyperactivated in MDD. In addition, this modulation may also have beneficial effects on the amygdala's activity, which would be involved in the self-dysregulation of the organisms along with reduced vagal control. With regard to this aspect, awe can stimulate the activation of the parasympathetic system and promote the withdrawal of the sympathetic one, thereby modulating the activity of the vagal nerve on the heart. Moreover, awe's self-transcendence nature would act as a counterpart to the incessant self-referential process at the base of rumination and sense of hopelessness. Awe acts as a trigger of accommodation, thereby fostering a process of positive change ranging from basic beliefs to more specific expectations of events. This potential of awe could be beneficial for overcoming cognitive fixedness and updating existing *prior* hypotheses used by people to predict and react to world circumstances.

## Conclusions: Potential for Awe-Based Interventions in MDD

Positive psychology's recent endeavors to understand the potential of specific discrete positive emotions for health promotion (e.g., Barrett-Cheetham et al., 2016) have partially involved awe. However, this complex emotion has shown great potential at several levels in the last 20 years. First, from this perspective, we proposed an up-to-date unifying proposal of awe's functioning, which allowed for a revision of all the empirical evidence supporting the potential therapeutic role of awe for contrasting specifically MDD. The core message of this work concerns the elicitation of awe as a potential therapeutic integrative intervention for contrasting depression.

Although awe induction has not been explicitly considered as a clinical intervention for depression, we reported evidence showing that even an experimental induction of awe had several beneficial effects for mental health, with some also overlapping specific depression components. Figure 2 shows all connections between awe and depression.

Recent research has demonstrated that the combination of format and content of a given awe-inspiring technique influenced the intensity of awe experience by participants (Chirico et al., 2017b), in order to enhance the ecological validity of the resulting experience. Therefore, it would be useful to focus not just on the most suitable “instrument” to elicit awe, but also on the content used to induce this emotion.

At the level of emotion-induction techniques, recent evidence has outlined different effective awe-eliciting techniques ranging from videos to virtual reality (VR) (Chirico et al., 2016). However, the long-term potential of videos, music, images, and VR for inducing awe has not been tested yet. Although their momentary benefits can be promising for administering doses of awe to people suffering from MDD and subthreshold conditions, a research protocol based on awe for MDD should assess the long-term effects associated to an awe experience as well as the durability of its benefits. Moreover, studies on dispositional awe have shown that a repeated exposure to this emotion brought forth benefits, especially at the hormonal level, which are just assumed to be durable (Stellar et al., 2015).

Moreover, given the social distancing measures adopted worldwide during the Covid-19 pandemic, another caveat would concern the possibility of delivering this intervention remotely as well. For instance, videos can be an effective, though less immersive and involving, solution to implement at distance. On the other hand, a VR setup, which provides an affordable and engaging tool, could be applied in a lab context following specific hygiene standards.

Another key aspect concerns the nature of the awe-inspiring *content* featured in images, videos, VR, and music. Given the multifaceted nature of this emotion, effective awe-inducing stimuli should provide an appropriate balance in terms of valence, thereby resulting into an emotionally mixed experience, which should be validated in advance to allow for the control over other potentially intervenient emotions (Chirico et al., 2017a) and could rely on individuals' preferences for specific elicitors. Combining more awe-eliciting techniques could be a potential solution able to enhance the effectiveness of awe-based interventions. For instance, customized self-selected awe-inspiring music (i.e., reflecting participants' preferences and personality) (Silvia et al., 2015) could be used in combination with visual techniques, such as images and VR, to enhance the personal relevance associated with an awe-inspiring intervention while always using standardized materials. A growing body of evidence concerns the potential of naturalistic scenarios able to release the self-transcendent nature of awe (Bethelmy and Corraliza, 2019) and nature itself, as one of awe's key elicitors (Ballew and Omoto, 2018; Graziosi and Yaden, 2019; Yaden et al., 2019), resulting in a powerful way to contrast depressive symptoms (e.g., Reklaitiene et al., 2014; Lopes et al., 2020) in both real and digital formats (Browning et al., 2020).

At this stage, we have outlined the key points to define the links between awe and MDD as well as guidelines for designing effective awe-based interventions for MDD. Extant evidence has depicted awe as an encounter with something greater and infinite that could don the guise of an explosion in terms of perceived possibilities, thereby acting as a new “big bang” (or new start) in our lives. This new overarching level would encompass all those that we have introduced so far (i.e., hormonal, neurophysiological, and psychological), acting as a spiral of change. Maybe this is also why awe has often been considered a core moment of transformation (Schneider, 2009). In this regard, the potential of awe for depression might also reside in this renewed sense of perceived *possibilities* (Chirico, 2020), which is at the basis of life and the need to trust the future. This sense would stem from neural processes and hormonal ones, reinforced by other persons and nature itself, while bridging our life, our world view, and sufferance, considering the complexity of life and the universe. All this complexity can be encapsulated in even a constrained space, such as a lab or room, if the specifics of this emotion are considered carefully and interventions are built upon them.

## Author Contributions

AC wrote the first draft and the final version. AC conceived the rational while AG revised and supervised the entire work.

## Conflict of Interest

The authors declare that the research was conducted in the absence of any commercial or financial relationships that could be construed as a potential conflict of interest.
